# Balloon Aortic Valvuloplasty Prior to Self‐Expanding TAVI: The BAVSE‐TAVI Registry

**DOI:** 10.1002/ccd.31731

**Published:** 2025-07-01

**Authors:** Abdalazeem Ibrahem, Ahmed Abdalwahab, Diana A. Gorog, Debbie Stewart, Rajiv Das, Richard Edwards, Mohaned Egred, Azfar Zaman, Mohammad Alkhalil, Mohamed Farag

**Affiliations:** ^1^ Cardiothoracic Department Freeman Hospital Newcastle Upon Tyne UK; ^2^ Centre for Health Services Research, School of Life and Medical Sciences University of Hertfordshire Hertfordshire UK; ^3^ Cardiovascular Department, Faculty of Medicine Tanta University Tanta Egypt; ^4^ National Heart and Lung Institute Imperial College London UK

**Keywords:** balloon aortic valvuloplasty, outcome, predilatation, TAVI, transcatheter heart valve

## Abstract

**Background:**

Direct transcatheter aortic valve implantation (TAVI) approach is feasible and safe compared to predilatation‐TAVI. Certain clinical and computerized tomography (CT)‐based characteristics might indicate the need for balloon aortic valvuloplasty (BAV) before TAVI, especially with self‐expanding valves.

**Aims:**

We aimed to identify patients who require predilatation before TAVI.

**Methods and Results:**

We performed a retrospective, single‐center study between 2020 and 2024, enrolling 315 patients (predilatation = 158 vs. direct = 157) aged 81 ± 6 years, 43.5% male, with EuroSCORE II of 3.7 ± 2.9. The rate of predilatation increased over the study period and was performed more often in patients with higher velocity and pressure gradients on echocardiography, higher aortic valve calcium score on CT, bicuspid morphology, bigger aortic annulus anatomy, severe aortic cusp calcification, tortuous descending aorta (bend > 60**°**), and horizontal ascending aorta (angle > 50**°**). Direct implantation was performed more frequently in patients with permanent pacemaker, ischemic heart disease, concomitant significant aortic regurgitation, or alternative‐access TAVI. Regression analysis demonstrated that only the horizontal aorta was an independent predictor of predilatation (*p* = 0.037). The rates of valve recapture, embolization, contrast use, procedure duration, hospital stay, inpatient death, stroke, significant paravalvular leak on postprocedural echocardiography, and new pacemaker implantation were not different between the groups. The rate of BARC ≥ 3 bleeding, mainly due to access‐site complications, was more frequent with direct‐TAVI compared to predilatation (6.4% vs. 0.6%; *p* = 0.005).

**Conclusions:**

Both predilatation and direct‐TAVI approaches can be safely performed in routine practice. Upfront selection of either approach based on the patient characteristics, echocardiography gradients, and CT anatomical features is recommended.

**Abbreviations**
BAV: balloon aortic valvuloplasty
CT: computerized tomography
IHD: ischemic heart disease
TAVI: transcatheter aortic valve implantation
THV: transcatheter heart valve

## 1 Introduction

Transcatheter aortic valve implantation (TAVI) is considered for patients with severe symptomatic aortic stenosis who are elderly or when surgical valve replacement is prohibitively high‐risk. Balloon aortic valvuloplasty (BAV) or predilatation before TAVI has been considered a mandatory step before valve deployment, as the clinical perception is that it eases transcatheter heart valve (THV) crossing, creates optimal space for adequate valve expansion and aortic‐annulus seal, and improves the overall procedural stability and haemodynamics [1]. However, with growing experience with the TAVI procedure and device evolution, predilatation is not routinely performed [2, 3].

Several real‐world registries and clinical trials, including three small randomized trials, have shown that the direct‐TAVI approach, without preceding BAV, in both self‐expanding and balloon‐expandable valves, is feasible and safe compared to the predilatation approach [3, 4]. Nonetheless, in routine practice, certain clinical and computerized tomography (CT)‐based characteristics might indicate the need for BAV before TAVI, especially with self‐expanding THVs in the setting of challenging anatomy.

Controversy also exists regarding the precise impact of predilatation in terms of simplifying the TAVI procedure and on complications, including stroke, paravalvular leak, and pacemaker implantation rates [5]. We aimed to perform a retrospective analysis of patients undergoing TAVI using a self‐expanding THV, with or without preceding BAV, at a large volume center to help identify clinical and/or anatomical features that can help indicate the need for predilatation before TAVI.

## 2 Methods

### 2.1 Study Design and Population

The BAVSE‐TAVI (Balloon Aortic Valvuloplasty before Self‐Expanding TAVI) was a single‐center, retrospective observational study. The study was conducted in accordance with the Declaration of Helsinki and Good Clinical Practice guidelines. Anonymized data were obtained from the institutional database normally utilized for patient care, and therefore, the need for formal informed consent/ethical approval was waived by the institutional review board. Anonymized demographic, echocardiographic, CT, operative, and outcome data prospectively collected, validated, and entered into the database were analyzed. All patients who underwent TAVI for severe symptomatic aortic valve stenosis with a self‐expanding valve (using Evolut PRO, PRO+ or FX, Medtronic, Minneapolis, MN) with or without predilatation were included. All cases were discussed and approved by the heart team. The study period was limited to procedures performed from January 2020 to June 2024, to align with relatively contemporary practice and technology. Patients who had predominant severe aortic valve regurgitation or valve‐in‐valve TAVI were excluded.

### 2.2 Imaging Modalities

Echocardiography was performed within 4 months of the TAVI procedural date and was reported according to British Society of Echocardiography guidelines [6]. ECG‐gated aortic valve calcium score and contrast‐enhanced CT aortic angiograms were conducted with ≥ 64 slice scanners. Scans were analyzed using 3mensio Aortic software, Pie Medical Imaging, The Netherlands, and reported by experienced physicians, according to the Society of Cardiovascular CT guidelines [7]. All TAVI procedures were performed at the Freeman Hospital, Newcastle Upon Tyne, UK, by five experienced physicians. All aspects of the TAVI procedure, including the choice of access route and predilatation were determined by the clinical team.

### 2.3 Study Outcomes

The primary study outcomes included all‐cause death, stroke, major vascular complications, major bleeding, significant paravalvular leak, or new permanent pacemaker implantation during hospitalization. The secondary study outcome was all‐cause death at midterm follow‐up. Stroke only included ischemic events confirmed on brain imaging with accompanying disability. Major vascular complications were defined using the Valve Academic Research Consortium‐2 (VARC‐2) criteria [8]. Major bleeding was defined as Bleeding Academic Research Consortium (BARC) type ≥ 3 [9]. Significant paravalvular leak was defined as more than a mild leak on postprocedural transthoracic echocardiography.

### 2.4 Statistical Analysis

Paired and unpaired *t*‐tests were used for comparison of normally distributed and Wilcoxon rank sum test used for non‐normally distributed variables. Dichotomous variables were compared using Fisher's exact test. Correlations were analyzed using Spearman's method. The ability of the echocardiographic and CT parameters to discriminate between patients with and without prior BAV was evaluated by the receiver‐operating characteristic (ROC) curve analysis. Univariate and multivariate linear regression analyses were used to identify factors associated with BAV before TAVI. The bootstrap technique using 1000 samples was used to account for the final multivariable model uncertainty. All study variables were first analyzed with univariate analysis and those that showed a significant interaction (*p* < 0.1) were entered into the final multivariable analysis. Analyses were performed with Stata V.15.1 (StataCorp, College Station, TX).

## 3 Results

### 3.1 Characteristics of the Study Population

**Figure 1:**
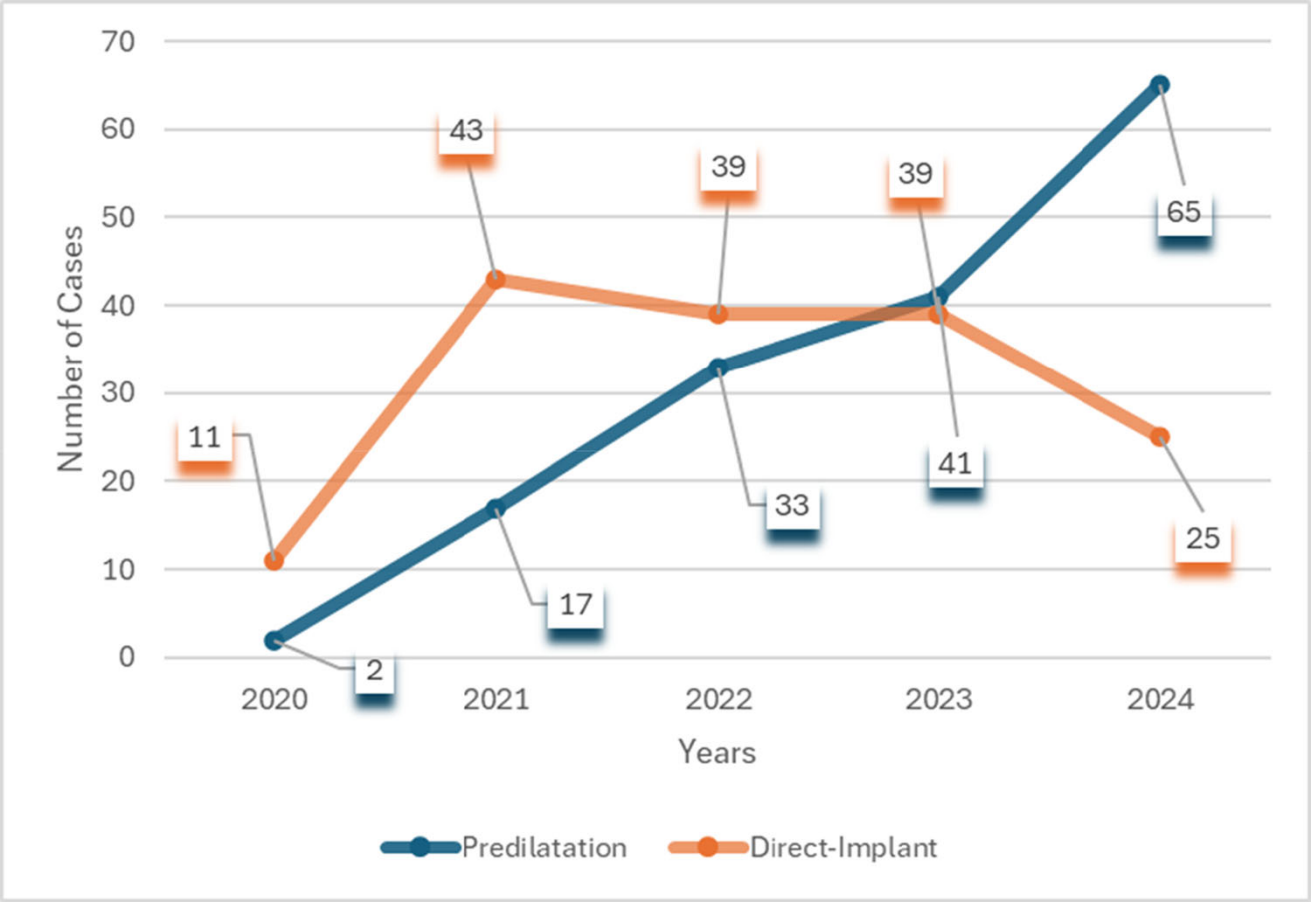
The rate of predilatation and direct implantation for transcatheter aortic valve implantation. The study period was limited to procedures performed from January 2020 to June 2024. [Color figure can be viewed at wileyonlinelibrary.com]

A total of 315 consecutive patients involving urgent and elective hospital admissions were included in the study. Predilatation was performed in 158 patients and direct implantation in 157 patients. The records and procedural scans/images of all patients were reviewed. Baseline patient characteristics are shown in Table 1. The groups were balanced in age, gender, and EuroSCORE II. Direct implantation was performed more frequently in patients with previous permanent pacemaker and ischemic heart disease (IHD). The rate of predilatation increased over the study period (Figure 1).

**Table 1:** Baseline patient characteristics.

	Whole group (*n* = 315)	Predilatation (*n* = 158)	Direct‐implant (*n* = 157)	*p* value
Age (years)	81 ± 6	81 ± 6	81 ± 6	0.475
Male	137 (43.5)	77 (48.7)	60 (38.2)	0.069
Body mass index (kg/m^2^)	27.9 ± 5.9	27.8 ± 5.8	28.1 ± 6.2	0.795
EuroSCORE II	3.7 ± 2.9	3.4 ± 2.4	4.0 ± 3.2	0.133
Previous pacemaker implantation	21 (6.7)	6 (3.4)	15 (9.5)	**0.044**
Previous cerebrovascular accident	25 (7.9)	13 (8.2)	12 (7.6)	1.000
Previous ischemic heart disease	102 (32.4)	39 (24.7)	63 (40.1)	**0.004**
Chronic kidney disease	157 (49.8)	74 (46.8)	83 (52.9)	0.311
Baseline electrocardiogram				
Atrial fibrillation	72 (22.8)	36 (22.8)	36 (22.9)	
LBBB	11 (3.5)	7 (4.4)	4 (2.5)	
RBBB	19 (6.0)	9 (5.6)	10 (6.4)	0.872
Implantation year				
2020	13 (4.1)	2 (1.3)	11 (7.0)	
2021	60 (19.0)	17 (10.8)	43 (27.4)	
2022	72 (22.9)	33 (20.9)	39 (24.8)	
2023	80 (25.4)	41 (25.9)	39 (24.8)	
2024^a^	90 (28.6)	65 (41.1)	25 (15.9)	n/a

*Note:* Values are presented as mean ± standard deviation or number (%). Chronic kidney disease is defined as an estimated glomerular filtration rate of < 60 mL/min. Bold values indicate statistically significant.

Abbreviations: LBBB, left bundle branch block; RBBB, right bundle branch block.

a The study period was limited to procedures performed up to June 2024.

### 3.2 Echocardiographic, CT, and Procedural Characteristics

In comparison to direct TAVI, predilatation was performed more frequently in patients with higher aortic valve peak velocity on echocardiography (4.5 ± 0.7 vs. 4.0 ± 0.6; *p* < 0.001), higher pressure gradients across the aortic valve on echocardiography (peak 83.6 ± 27.1 vs. 68.6 ± 20.2 mmHg, mean 51.2 ± 17.9 vs. 40.8 ± 13.7 mmHg; *p* < 0.001), higher aortic valve calcium score on CT (3239 ± 1976 vs. 1902 ± 1371 Agatston units; *p* < 0.001), bicuspid morphology, bigger aortic annulus anatomy, severe aortic cusp calcification, tortuous descending aorta (bend > 60**°**), and horizontal ascending aorta (angle > 50**°**). Patients with predilatation required more frequent post‐dilatation than those with direct implantation (15.2% vs. 3.8%; *p* < 0.001). There was a positive correlation between post‐dilatation and aortic valve calcium score on CT (*r* = 0.4, *p* < 0.001), bicuspid morphology (*r* = 0.4, *p* < 0.001), and severe aortic cusp calcification (*r* = 0.3, *p* < 0.001). Direct implantation was performed more frequently in patients with concomitant significant aortic regurgitation and alternative‐access TAVI (Table 2).

**Table 2:** Echocardiographic, computerized tomography, and procedural characteristics.

	Whole group (*n* = 315)	Predilatation (*n* = 158)	Direct‐implant (*n* = 157)	*p* value
Aortic valve peak velocity (m/s)	4.3 ± 0.7	4.5 ± 0.7	4.0 ± 0.6	**< 0.001**
Aortic valve area (cm^2^)	0.7 ± 0.2	0.7 ± 0.2	0.8 ± 0.2	0.197
Aortic valve area index (cm^2^/m^2^)	0.4 ± 0.1	0.3 ± 0.1	0.4 ± 0.1	**0.034**
Aortic valve peak gradient (mmHg)	76.0 ± 24.9	83.6 ± 27.1	68.6 ± 20.2	**< 0.001**
Aortic valve mean gradient (mmHg)	45.9 ± 16.8	51.2 ± 17.9	40.8 ± 13.7	**< 0.001**
Left ventricular ejection fraction (%)	51.5 ± 9.9	52.3 ± 9.0	50.6 ± 10.8	0.113
Concomitant significant aortic regurgitation^a^	8 (2.5)	0 (0)	8 (5.1)	**0.003**
Aortic valve calcium score (Agatston units)	2562 ± 1821	3239 ± 1976	1902 ± 1371	**< 0.001**
Bicuspid aortic valve morphology	8 (2.5)	7 (4.4)	1 (0.6)	**0.034**
Maximum aortic annulus diameter (mm)	26 ± 3	27 ± 3	26 ± 3	**0.006**
Minimum aortic annulus diameter (mm)	20 ± 3	20 ± 2	19 ± 3	**0.005**
Aortic annulus area (mm^2^)	427 ± 90	446 ± 89	408 ± 87	**< 0.001**
Aortic annulus perimeter (mm)	74 ± 8	76 ± 9	73 ± 8	**0.012**
Severe aortic cusp calcification^b^	51 (16.2)	34 (21.5)	17 (10.8)	**0.014**
Left coronary artery height (mm)	14 ± 3	15 ± 3	14 ± 3	0.108
Right coronary artery height (mm)	16 ± 3	17 ± 3	16 ± 3	**0.025**
Tortuous descending aorta (bend > 60°)	8 (2.5)	7 (4.4)	1 (0.6)	**0.034**
Horizontal aorta (angle > 70°)	9 (2.8)	6 (3.8)	3 (1.9)	0.330
Horizontal aorta (angle > 50°)	119 (37.8)	74 (46.8)	45 (28.7)	**< 0.001**
Duration of hospital stay (days)	3 ± 6, 1 (1–2)^c^	3 ± 6	3 ± 6	0.861
Transfemoral access	310 (98.4)	158 (100)	152 (96.8)	**0.040**
Post‐dilatation	30 (9.5)	24 (15.2)	6 (3.8)	**0.001**
Implanted valve size (mm)				
23	15 (4.8)	11 (7.0)	4 (2.5)	0.109
26	121 (38.4)	67 (42.4)	54 (34.4)	0.165
29	137 (43.5)	64 (40.5)	73 (46.5)	0.307
34	42 (13.3)	16 (10.1)	26 (16.6)	0.100
Number of recaptures during implantation > 3	13 (4.1)	8 (5.0)	5 (3.2)	0.573
Second valve needed due to valve embolization	4 (1.2)	2 (1.2)	2 (1.2)	1.000
Contrast volume (mL)	99 ± 47	101 ± 48	97 ± 45	0.377
Procedural length (min)	81 ± 31	79 ± 26	83 ± 36	0.238

*Note:* Values are presented as mean ± standard deviation or number (%). Bold values indicate statistically significant.

^a^ Combined significant aortic regurgitation is defined as more than or equal to moderate aortic regurgitation on echocardiography.

^b^ Severe aortic cusp classification is defined as heavy circumferential calcification in all aortic cusps.

^c^ Median values (Q1–Q3).

**Figure 2:**
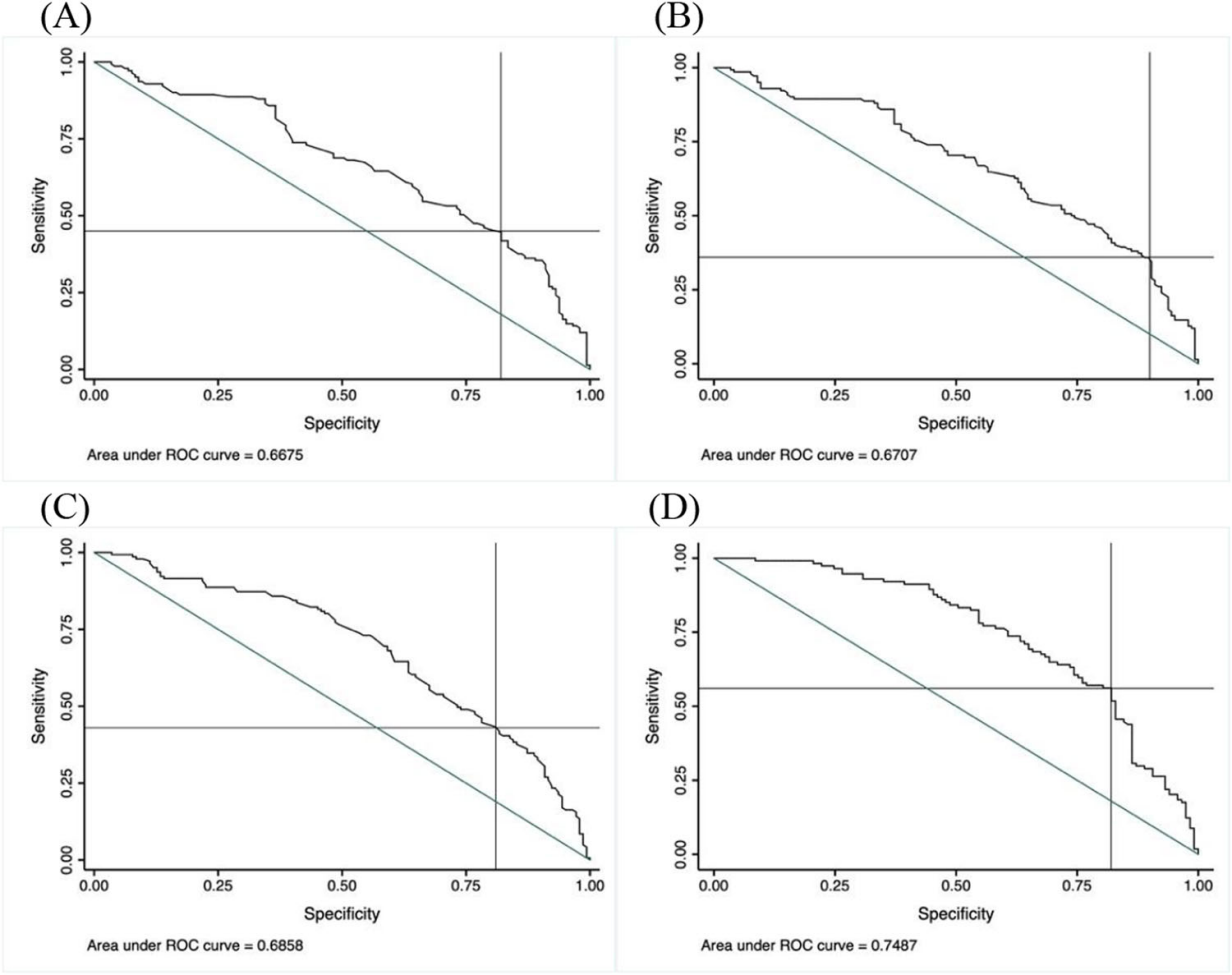
Receiver‐operating characteristic curves for (A) peak aortic valve velocity on echocardiography, (B) peak aortic valve pressure gradient, (C) mean aortic valve pressure gradient, and (D) aortic valve calcium score on CT, for predilatation before transcatheter aortic valve implantation. Peak aortic valve velocity ≥ 4.5 m/s, peak pressure gradient ≥ 90 mmHg, mean pressure gradient ≥ 50 mmHg, and aortic valve calcium score ≥ 2704 Agatston units were the optimal cut‐points to predict predilatation before TAVI, with sensitivity 45% and specificity 82%, sensitivity 36% and specificity 90%, sensitivity 43% and specificity 81%, and sensitivity 56% and specificity 82%, respectively.

Peak aortic valve velocity ≥ 4.52 m/s, peak pressure gradient of ≥ 90.2 mmHg, and mean pressure gradient of ≥ 50.1 mmHg were the optimal cut‐points to predict the use of BAV, with sensitivity 45% and specificity 82%, sensitivity 36% and specificity 90%, and sensitivity 43% and specificity 81%, respectively (Figure 2A−C). Likewise, aortic valve calcium score ≥ 2704 Agatston units was the optimal cut‐point to predict BAV, with sensitivity 56% and specificity 82% (Figure 2D), and no significant difference according to gender.

The rates of valve recapture, embolization with a second valve needed, contrast volume use, and procedural length were not different between the groups (Table 2).

### 3.3 In‐Hospital Outcomes

The duration of hospital stay and the occurrence of inpatient death, stroke, major vascular complications (VARC‐2 criteria), significant paravalvular leak, and new permanent pacemaker implantation were not different between the groups (Tables 2 and 3). The rate of BARC ≥ 3 bleeding was more frequent with direct‐TAVI compared to predilatation (6.4% vs. 0.6%; *p* = 0.005). All bleeding events, apart from one requiring pericardiocentesis, were due to femoral access‐site complications.

**Table 3:** Inhospital clinical outcomes.

	Whole group (*n* = 315)	Predilatation (*n* = 158)	Direct‐implant (*n* = 157)	*p* value
Death	9 (2.8)	6 (3.8)	3 (1.9)	0.501
Ischemic stroke	14 (4.4)	8 (5.0)	6 (3.8)	0.786
Major vascular complications (VARC‐2)	6 (1.9)	1 (0.6)	5 (3.2)	0.121
Major bleeding (BARC ≥ 3)	11 (3.5)	1 (0.6)	10 (6.4)	**0.005**
Significant paravalvular leak^a^	18 (5.7)	11 (6.9)	7 (4.4)	0.468
New permanent pacemaker implantation	35 (11.1)	16 (10.1)	19 (12.1)	0.596

*Note:* Bold value indicates statistically significant.

Abbreviations: BARC, Bleeding Academic Research Consortium; VARC‐2, Valve Academic Research Consortium‐2.

a Defined as more than a mild leak on postprocedural transthoracic echocardiography.

Of all the characteristics in Tables 1, 2, 3, the following were associated with predilatation using univariate regression analysis: pacemaker‐naïve patients, IHD‐naïve patients, high aortic valve velocity, small aortic valve area, higher aortic valve gradients, lack of significant aortic regurgitation, higher aortic valve calcium score, bicuspid morphology, bigger aortic annulus diameters, area and perimeter, severe aortic cusp calcification, tortuous descending aorta, horizontal ascending aorta (angle > 50**°**), post‐dilatation, and lower major bleeding rates. Multivariate regression analysis demonstrated that only the horizontal aorta (angle > 50**°**) was an independent predictor of the need for predilatation (*p* = 0.037).

### 3.4 Secondary Outcome

At a medium follow‐up of 2 (1−3) years, there was no difference in all‐cause death between direct‐TAVI and predilatation (31.2% vs. 23.4%, respectively; *p* = 0.131).

## 4 Discussion

The main finding of this study is that both predilatation and direct‐TAVI approaches were shown to be similarly safe in routine practice. The rates of valve recapture, embolization, contrast use, procedural length, hospital stay, inpatient death, stroke, significant paravalvular leak on postprocedural echocardiography, and new pacemaker implantation were not different between the groups. Also, midterm all‐cause mortality was not different between the groups. The rate of major bleeding due to femoral vascular complications was significantly more frequent with the direct‐TAVI approach compared to predilatation.

Predilatation before TAVI with a self‐expanding valve has gained more focus in recent years, especially in heavily calcified valves, to optimize the THV deployment. In the BHF PROTECT‐TAVI trial, approximately 43% of patients had a self‐expanding valve, with around 45% of the study population requiring predilatation [10]. In the DEDICATE‐DZHK6 trial, 35% of patients had a self‐expanding valve, with around 49% of the study population requiring predilatation [11]. In the SMART trial, predilatation was more common in the self‐expanding group (41.8%) compared to the balloon‐expandable group (21.4%), with the former requiring more post‐dilatation procedures [12]. In the Evolut Low‐Risk trial, predilatation was performed in 34.9% of patients and post‐dilatation was performed in 31.3% of patients [13]. In a recent, large real‐world registry of 3353 patients undergoing TAVI with a self‐expanding valve, predilatation was performed in 53.5% of patients and post‐dilatation was performed in 31.8% of patients [14].

**Figure 3:**
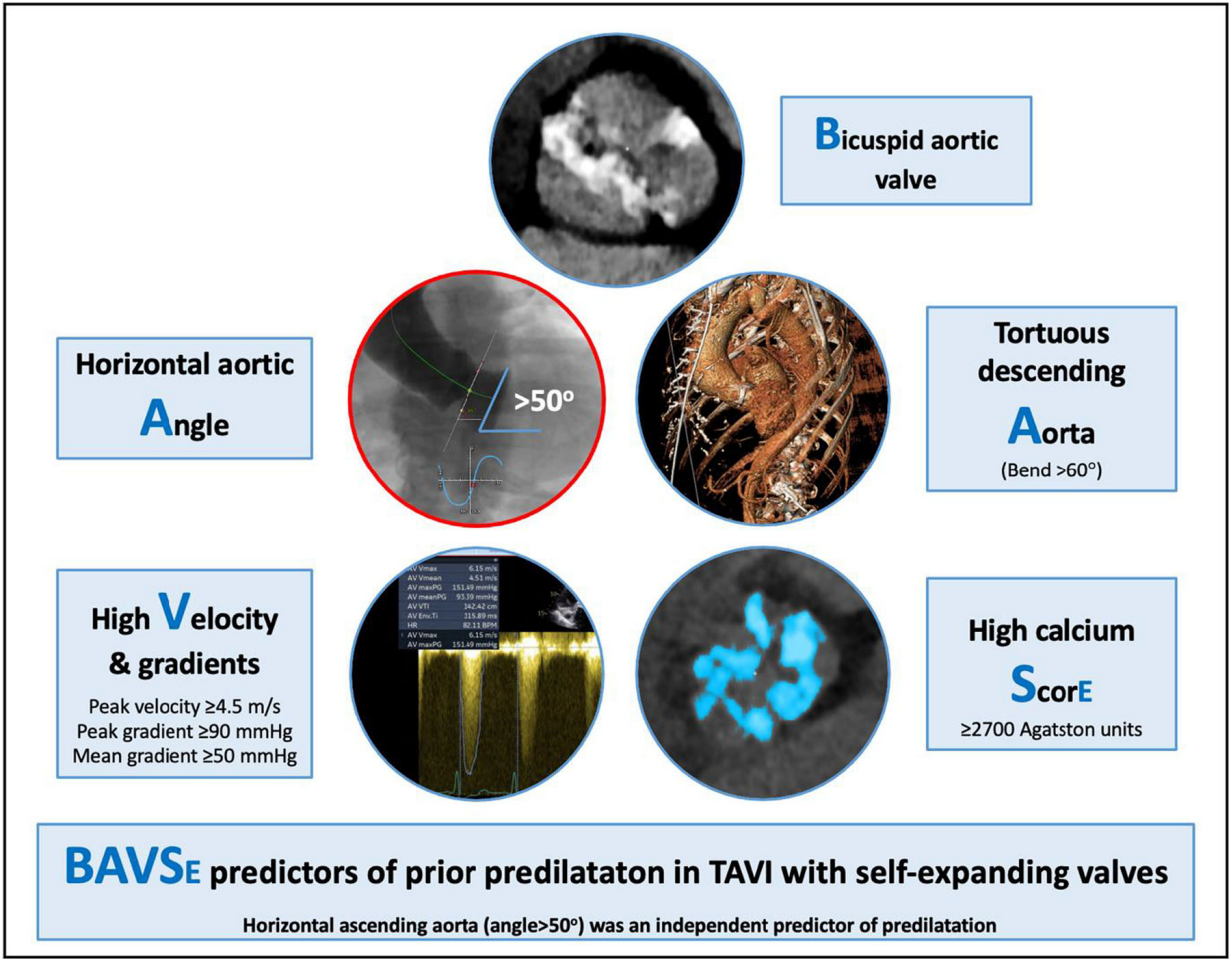
Central Illustration: The BAVSE registry predictors of predilatation before transcatheter aortic valve implantation with self‐expanding valves. Predilatation was performed more frequently in patients with higher aortic valve peak velocity and pressure gradients on echocardiography, higher aortic valve calcium score on CT, bicuspid morphology, tortuous descending aorta, and horizontal ascending aorta compared to direct implantation. [Color figure can be viewed at wileyonlinelibrary.com]

In our registry, predilatation was performed in about 50% of patients and post‐dilatation was required in 9.5%. Predilatation before TAVI was performed more in patients with higher pressure gradients on echocardiography, higher aortic valve calcium score on CT, bicuspid morphology, bigger aortic annulus anatomy, severe aortic cusp calcification, tortuous descending aorta, and horizontal ascending aorta (angle > 50**°**) (Figure 3). Regression analysis demonstrated that only the horizontal ascending aorta was an independent predictor of predilatation. Theoretically, predilatation before TAVI facilitates THV crossing and deployment, as it reduces the possibility of technical challenges, which may occur with very tight calcified valves with fused commissures. Importantly, predilatation helps mitigate the haemodynamic instability that happens with the dynamic positioning of the self‐expanding THV through a tight non‐dilated orifice, which may precipitate transient flow obstruction. Moreover, predilatation of tight calcified native valves may negate any under‐expansion of the self‐expanding THV, resulting in a significant paravalvular leak and increasing the embolization risk. Additionally, enhanced haemodynamic instability while deploying the THV through tight and calcific valves may prolong cerebral hypoperfusion, which may precipitate a neurological insult. This phenomenon is more often encountered with self‐expanding, compared to balloon‐expandable valves, as the deployment process usually takes longer during THV unsheathing steps [15]. Use of relatively small balloons for predilatation might reduce the incidence of permanent pacemaker requirement after THV implantation [16]. This can be achieved by using balloons smaller than 23 mm and/or a balloon‐diameter to aortic annulus‐diameter ratio of 0.65 [1]. In our cohort, patients with predilatation required more frequent post‐dilatation than those with direct implantation, probably reflecting the complexity of the valve anatomy, as post‐dilatation significantly correlated with calcified and/or bicuspid aortic valves.

Direct implantation was performed more in patients with permanent pacemakers, IHD, concomitant significant aortic regurgitation, and alternative‐access TAVI. One of the proposed advantages of the direct‐TAVI approach is the reduction of periprocedural ischemic stroke by reducing the risk of degenerated valve debris embolization after balloon dilatation [15]. Nonetheless, our study aligns with most TAVI studies showing no significant difference in stroke incidence with predilatation versus direct implantation [17]. Pacemaker requirements after TAVI were lower with direct implantation; however, our study did not demonstrate an increased risk of pacing with predilatation versus direct implantation [18]. Direct TAVI implantation was significantly associated with major access‐site bleeding, which is probably due to the play of chance, as several studies did not demonstrate this association. However, it is possible that patients who had direct‐TAVI did not receive proper serial dilatations of the femoral access site with larger than 5−6 Fr sheaths to facilitate BAV, perhaps contributing to the direct sheathless delivery‐system nose‐cone injury of the Medtronic Evolut valve.

### 4.1 Study Limitations

This was a single‐center, retrospective observational study and therefore, has inherent limitations of the retrospective design. Unit and technical expertise within the team, as well as valve technology, improved over time. Nevertheless, this study highlights the real‐world experience of when to perform predilatation before TAVI based on clinical, echocardiographic, and CT characteristics. Second, the results only apply to TAVI using the Medtronic Evolut self‐expanding THVs. Other valve platforms with low opening force may mandate routine upfront predilatation before TAVI. Third, our analysis demonstrated that the rate of BARC type ≥ 3 major bleeding due to femoral vascular complications was significantly more frequent with the direct‐TAVI approach compared to predilatation. However, the VARC‐2‐defined major vascular complications were not significant between the groups. Discrepancies between BARC and VARC‐2 can occur when a BARC type 3−5 bleeding event does not meet the VARC‐2 criteria for a major vascular event. For example, a BARC type 3a bleed requiring a transfusion but not necessarily requiring intervention might not be classified as major by VARC‐2. Fourth, our registry lacks data on left ventricular outflow tract calcification. Pre‐ and post‐dilatation rates are higher in those patients with moderate/severe outflow tract calcification. Fifth, the decision of predilatation was made by the operator, with resultant selection bias. Subsequently, the results may only reflect our center's practice and may not be generalizable to the wider population. However, to date, the choice of predilatation before TAVI remains a largely subjective decision based on the experience of the center and operator, and our study provides more insight into when this might be useful to perform. The increase in the rate of predilatation over the study period reflects the complexity of the anatomies treated in recent years with advances in procedural techniques, valve technology, institutional, and operator experience. Our center's predilatation rate remains around 50% for all TAVI procedures using the Medtronic Evolut self‐expanding THV, mainly due to its high opening force. Finally, our midterm analysis reports all‐cause mortality, and thus deaths unrelated to the TAVI procedure might have been included. Having said that, all‐cause mortality is considered an appropriate endpoint to follow in the long term because it accounts for both cardiac and systemic diseases and is unaffected by any reporting or misclassification bias.

## 5 Conclusions

Both predilatation and direct‐TAVI approaches are frequently performed in routine practice and appear equally safe. An upfront selection of either approach based on the patient characteristics and anatomy is recommended. In patients undergoing TAVI with a self‐expanding valve, there is a group that requires BAV before valve implantation to improve procedural success and reduce peri‐procedural complications. Horizontal ascending aorta (angle > 50**°**) was an independent predictor of predilatation in our study. Further studies incorporating detailed anatomical and haemodynamic assessments are needed to help clinicians choose the right strategy for self‐expanding THV implantation.

## Conflicts of Interest

The authors declare no conflicts of interest.
